# Effects of the Ratio of Alaskan Pollock Surimi to Wheat Flour on the Quality Characteristics and Protein Interactions of Innovative Extruded Surimi–Flour Blends

**DOI:** 10.3390/foods14162851

**Published:** 2025-08-17

**Authors:** Xinru Fan, Xinyue Zhang, Yingying Zhou, Maodong Song, Meng Li, Soottawat Benjakul, Zhibo Li, Qiancheng Zhao

**Affiliations:** 1 College of Food Science and Engineering, Dalian Ocean University, Dalian 116023, China; 2 Dalian Key Laboratory of Marine Bioactive Substances Development and High Value Utilization, Dalian 116023, China; 3 Liaoning Provincial Marine Healthy Food Engineering Research Centre, Dalian 116023, China; 4 International Center of Excellence in Seafood Science and Innovation, Faculty of Agro-Industry, Prince of Songkla University, Hat Yai 90110, Thailand

**Keywords:** wheat flour, Alaskan pollock surimi, blending ratio, extrusion, expansion rate

## Abstract

Snack foods (e.g., extruded flour-based products) are widely favored by consumers because of their convenience, affordability, and time-saving attributes. However, with the growing demand for high-quality snacks, several challenges have emerged that hinder industry development, such as relatively underdeveloped industrial standards, limited raw material diversity (primarily starch and soy protein), and, consequently, insufficient nutritional value. In this study, a novel type of puffed snack was developed using Alaskan pollock surimi and wheat flour using extrusion puffing technology. The effects of varying ratios of surimi to wheat flour (0:10, 1:9, 2:8, 3:7, and 4:6, which served as SFBC, SFB1, SFB2, SFB3, and SFB4, respectively), on the physicochemical properties, apparent morphology, microstructure, thermal stability, and protein structure of spicy strips were systematically investigated, and the interaction between extruded protein and flour mixtures was analyzed. The results indicated that increasing the proportion of surimi led to decreases in hardness, elasticity, and chewiness, whereas the moisture content and water solubility index increased. The maximum expansion rate (202.2%) was observed in the SFB1 sample. Morphological and microstructural observations further revealed that a higher surimi content resulted in a denser internal structure and a reduced degree of puffing. The protein distribution was relatively uniform, with large pores. Moreover, increased surimi content increased the proportion of immobilized water and improved the thermal stability. These findings provide valuable insights into starch–protein-complex-based extrusion puffing technologies and contribute to the development of innovative surimi-based puffed food products.

## 1. Introduction

With the acceleration of modern life and the gradual reduction in meal times, nutritious and delicious snack foods have become increasingly popular among consumers. Emerging snacks, particularly extruded puffed products, have gained a competitive edge in the food market because of their convenience, affordability, long shelf-life, portability, and time-saving attributes [1]. As an innovative processing technique, extrusion puffing has been widely adopted in the food industry because of its advantages, such as high energy efficiency, broad raw material adaptability, multifunctionality, and low production costs, leading to rapid development [2]. Puffed foods can be categorized on the basis of raw materials into starch-based, protein-based, fruit-based, vegetable-based, starch–plant protein mixed, and starch–animal protein mixed types. Currently, most commercial puffed snacks are starch-based varieties, such as spicy strips. As a typical starch-based puffed product, spicy strips are produced from wheat flour through extrusion and puffing processes and offer substantial economic benefits [3]. However, with the rapid growth of the food market, quality issues associated with pure starch-based puffed foods, including nutritional monotony, increased hardness, and diminished flavor, which significantly compromise the overall product quality, have increasingly surfaced [4].

Research indicates that ideal healthy snacks should contain higher levels of vitamins, dietary fiber, and protein, but have lower fat, sodium, and sugar contents [1]. To address these challenges and diversify puffed food offerings, researchers have explored the coextrusion of multiple raw materials and the development of novel puffed products. For example, incorporating hydrophilic colloids, dietary fiber, and protein into flour-based formulations has been proposed to increase nutritional value and expand product variety [5]. Nevertheless, high-protein formulations tend to produce a dense puffing structure with poor expansion, negatively affecting texture. Consequently, few products rely solely on protein for puffing [6]. In recent years, efforts have focused on developing starch–protein-blended extruded puffed foods. Studies involving the extrusion of rapeseed meal with varying protein contents (10–40%) and potato starch revealed that increasing rapeseed protein levels significantly decreased the cross-sectional expansion ratio and water absorption index [7]. Compared with research on single-component puffed foods such as starch-based snacks, studies on mixed puffed products remain limited in both quantity and depth. Most current investigations focus on optimizing basic process parameters and simple formulation adjustments and lack innovation and in-depth exploration of structural and physicochemical changes induced by extrusion. Therefore, future research on starch–protein mixed extruded puffed foods should evolve from fundamental material and process studies toward more comprehensive investigations into component interactions, influence mechanisms, and functional action pathways.

Surimi is produced from rinsed fish mince and is rich in myofibrillar proteins, to which cryoprotectants are mixed to facilitate long-term storage and convenient transportation [8]. Surimi-based products are manufactured through a series of processes, such as grinding, molding, and thermal denaturation, with the addition of various ingredients. These products have a long history of production, processing, and consumption in Asia regions [9]. Currently, the demand for raw materials for processing is increasing gradually. Previous studies have shown that incorporating silver carp paste into puffed food can not only increase its nutritional value but also inhibit starch hydrolysis by coating starch granules with fish protein, thus improving the overall nutritional and health profile of flour-based products [10]. Furthermore, adding surimi to wheat flour to increase its nutritional value has been demonstrated to be feasible. However, with increasing the surimi addition ratio, the product quality tends to decline significantly, leading to deterioration in taste, flavor, quality, and shape [11]. Therefore, identifying appropriate strategies to maximize the nutritional benefits of surimi while minimizing its negative impact on product characteristics is crucial for advancing research and development in related industries. Against the backdrop of increasing consumer awareness regarding health and sustainability, there is a growing demand for innovative high-protein products. Alaskan pollock surimi, characterized by its low fat and cholesterol content as well as high protein levels, has been widely utilized in the production of surimi-based products due to its abundant availability and cost-effectiveness [12]. It serves as an ideal ingredient for the addition of novel extruded flour-based products.

In this study, Alaskan pollock surimi was incorporated at different ratios (0:10, 1:9, 2:8, 3:7, and 4:6) to investigate its effects on extruded flour products. The physical properties of these products were explored using the moisture content, water absorption index, water solubility index, oil absorption capacity, expansion rate, textural properties, etc. Additionally, the structural characteristics of extruded samples were observed using their apparent and cross-sectional microstructures. In addition, the thermodynamic properties and moisture distributions of the samples were investigated by DSC, XRD, and low-field nuclear magnetic resonance. Finally, the protein secondary structure and SDS-PAGE were analyzed to express the changes in the protein composition and conformation. This study illustrates the influence of surimi addition on the quality of extruded flour-based products and determines the cross-linking of the wheat flour and surimi system. These results provide a better understanding of the role of surimi in extruded flour products and offer a scientific basis for the in-depth application of new puffed foods in the food industry.

## 2. Materials and Methods

### 2.1. Materials

Frozen Alaskan pollock (*Gadus chalcogrammus*) surimi (grade A, moisture content 70%) was supplied by Dalian Rich Food Co., Ltd. (Dalian, China). Wheat flour (Hetao household powder, protein 13.4 g/100 g, fat 1.4 g/100 g, carbohydrate 71.2 g/100 g) was obtained from Inner Mongolia Hengfeng Group Yinliang Flour Industry Co., Ltd. (Bayannur, Nei Mongol Autonomous Region, China). Canola oil was purchased from Yihai Kerry Arawana Holdings Co., Ltd. (Shanghai, China). Other chemical agents were of analytical grade.

### 2.2. Sample Preparation

The pre-thawed surimi (4 °C, overnight) was ground for 5 min (MJLZ235; Midea Group Co., Ltd., Foshan, China) and mixed with flour powder at ratios of 0:10 (control group), 1:9, 2:8, 3:7, and 4:6 (g:g). Moderate NaCl and water were added to the raw surimi-flour blended samples, and the final moisture content was adjusted to 30% (wet weight). The specific formula of the raw surimi flour blended (SFB) samples is presented in Table S1. The blended samples were subsequently transferred into a single-screw extrusion puffing machine (60 code, Guangju Machinery Manufacturing Factory, Xingtai, China) with a rotation speed of 720 r/min to finish the series process of maturation (180 °C), pressurization, and puffing. The five groups of extruded samples, after equilibration to room temperature (RT), were trimmed and stored in a refrigerator for further measurement and labeled SFBC, SFB1, SFB2, SFB3, and SFB4.

### 2.3. Moisture Content, Water Absorption Index (WAI), and Water Solubility Index (WSI)

The moisture content of the extruded SFB samples was measured following the methods of Liu et al. [13]. Approximately 2.0 g of each sample was placed into an evaporating dish and subsequently dried in a preheated oven at 105 °C until a constant weight. Each group was analyzed in triplicate. The results were calculated as follows:

$$Moisture\;Content\%=\frac{\left(W_{1}\right.-\left.W_{2}\right)}{W_{1}}\times 100\%$$

where *W*_1_ is the initial weight (g) of the extruded SFB, and *W*_2_ is the constant weight after the drying process.

The *WAI* and *WSI* were determined using Kaur et al.’s method [14]. Freeze-dried extruded SFB samples were ground, sieved (0.18 mm mesh), and collected. Approximately 0.25 g of SFB powder (*m*_1_) was added to 5 mL tubes (*m*_2_) and uniformly mixed with 3 mL of distilled water. The tubes containing the mixtures were incubated at 30 °C in a water bath for 30 min with 10 min intervals of shaking (45 s). The mixture was separated through centrifugation (1531× *g*, 20 min, RT), where the supernatant was collected in a weighing bottle (*m*_3_) at 105 °C and dried to a constant weight (*m*_4_). The weight of the centrifugal sediment and tube was recorded as *m*_5_. The *WAI* and *WSI* were computed as follows:

$$WAI\%=\frac{\left(m_{5}\right.-\left.m_{2}\right)}{m_{1}}\times 100\%$$

$$WSI\%=\frac{\left(m_{4}\right.-\left.m_{3}\right)}{m_{1}}\times 100\%$$

### 2.4. Oil Absorption Capacity (OAC)

The *OAC*s of extruded SFB samples were estimated on the basis of a slightly modified method from Bhinder et al. [15]. In brief, extruded SFB samples (2 g, *w*_1_) and rapeseed oil (20 mL) were mixed for 30 min in a 50 mL centrifuge tube with shaking for 10 min intervals (30 s). After oil absorption was complete, the surfaces of the samples were wiped with filter paper. The samples (*w*_2_) were subsequently centrifuged at 6873× *g* for 20 min to remove the remaining oil. The *OAC* was determined by the following formula:

$$OAC\%=\frac{\left(w_{2}\right.-\left.w_{1}\right)}{w_{1}}\times 100\%$$

### 2.5. Radial Expansion Rate (RER)

The *RERs* of extruded SFB samples were measured by a previously described method with slight modifications [1]. The internal and external diameters of the extruded SFB samples (*d*_1_, *d*_2_) and metal molds (*D*_1_, *D*_2_) were obtained from five measurement points for each individual sample. The radial expansion rate was determined using the following equation:

$$RER\%=\frac{\left(d_{2}\right.-\left.d_{1}\right)}{\left(D_{2}\right.-\left.D_{1}\right)}\times 100\%$$

### 2.6. Sensory Analysis

The sensory properties of all the samples were evaluated by 10 panelists who were students (between 20 and 24 years old) majoring in food science [8]. All panelists participated in the sensory evaluation course training. The samples were placed on white plates at RT and randomly presented to the panelists. The odor, color, hole distribution, appearance, and mouthfeel of all the samples were evaluated with assigned weights of 0.1, 0.2, 0.2, 0.2, and 0.3, respectively.

### 2.7. Color Properties

The surface of extruded SFB samples was detected by a colorimeter (Konica Minolta, CR-400, Tokyo, Japan) to estimate the L*, a*, and b* values, respectively. *Hue*, *chroma*, and color difference (∆*E*) were calculated as previously reported equations [14]. Each group of samples was tested three times. The whiteboard was regarded as the blank for the ∆*E* calculation, where *L*_0_*, *a*_0_*, and *b*_0_* were 77.69, 82.57, and 87.92, respectively.

$$Hue=arctan\frac{b^{*}}{a^{*}}$$

$$Chroma=\sqrt{a^{{*}^{2}}+b^{{*}^{2}}}$$

$$\Delta E=\sqrt{{\left(L^{*}-L_{0}^{*}\right)}^{2}+{\left(a^{*}-a_{0}^{*}\right)}^{2}+{\left(b^{*}-b_{0}^{*}\right)}^{2}}$$

### 2.8. Textural Properties

Texture profile analysis (TPA) was applied to determine the textural properties of the extruded SFB samples via a texture analyzer (TMS-PRO, Food Technology Corporation, Sterling, VA, USA) [16]. Five middle sections of extruded SFB samples were randomly selected from each group to perform the TPA test. The settings were as follows: P/5 probe, test speed of 1.5 mm/s, compression ratio of 65%, and trigger force of 5.0 g.

### 2.9. Microstructure Observation

#### 2.9.1. Optical Microscopy (OM)

The extruded SFB samples were prepared as 8 μm frozen sections using a Leica cryostat microtome (CM1950, Jena, Germany). The sections of each sample were subsequently stained with iodine–potassium iodide staining solution and observed at 200× magnification with a Nikon ECLIPSE Ci light microscope (Tokyo, Japan).

#### 2.9.2. Scanning Electron Microscopy (SEM)

The freeze-dried extruded SFB samples were broken in liquid nitrogen. The cross-section of each sample was sprayed with gold and observed under an S4800 SEM (Hitachi High-Tech Corporation, Tokyo, Japan) at 10.0 kV and 50× magnification [1].

### 2.10. Differential Scanning Calorimetry (DSC)

The thermal stability of extruded SFB samples was determined using the method of Lapčíková et al. [17]. Briefly, 5 mg of freeze-dried sample powder was placed in an aluminum crucible for detection via a differential scanning calorimeter (T20, TA Instruments, New Castle, DE, USA), where an empty aluminum crucible was used as the reference. The experimental parameters were set as follows: the heating range was from 50 °C to 230 °C, the heating rate was 15 °C/min, and the flow rate of the carrier gas (nitrogen) was 40 mL/min. The initial temperature (T_0_), peak temperature (T_p_), and enthalpy change (∆H) were recorded and calculated using an instrument to evaluate the thermal stability of the samples.

### 2.11. X-Ray Diffraction (XRD)

The X-ray patterns of the freeze-dried extruded SFB powder were detected using an X-ray diffractometer (D8 DISCOVER, Bruker Corporation, Karlsruhe, Germany). The results were collected through a Cu Kα source in the scanning range of 4 to 50°, where the scanning rate was 2°/min [18].

### 2.12. Low-Field Nuclear Magnetic Resonance (LF-NMR) and Magnetic Resonance Imaging (MRI)

The determination of the transverse relaxation time (*T*_2_) of the extruded SFB samples was performed in our previous study [19]. Briefly, the samples were adjusted to the Carr–Purcell–Meiboom–Gill sequence with the following settings: SF = 21 MHz, TW = 2000 ms, NS = 16, TD = 240,034, TE = 0.3 ms, and NECH = 4000. The proton density map of these samples was subjected to MRI using the same LF-NMR analyzer (MesoMR23-060 V-I, Niumag Electric Corporation, Suzhou, China). [20]

### 2.13. Secondary Structural Changes

The freeze-dried extruded SFB samples were ground and mixed with potassium bromide at a ratio of 1:100 (g:g). The spectral range from 4000 to 400 cm^−1^ was detected by FTIR spectroscopy (ALPHA II; Bruker, Karlsruhe, Germany). Peak Fit (version 4.12) was applied to deconvolute the range of 1700–1600 cm^−1^, and second-order fitting was performed to calculate the secondary structure content [9].

### 2.14. SDS–PAGE

The determination of SDS–PAGE patterns followed the methods of a previous study with slight adjustments [21]. Briefly, twenty grams of freeze-dried extruded SFB samples were dissolved in 200 μL of loading buffer (5×) overnight and centrifuged at 1531× *g* for 10 min to collect the supernatant. For each lane, 10 μL of the supernatant was loaded on a 5% concentrated gel and a 12% separation gel.

### 2.15. Statistical Analysis

All the experiments were conducted in triplicate, and the results are presented as the means ± SDs. The statistical analyses were performed using SPSS (version 26.0, SPSS Inc., Chicago, IL, USA) analysis software, which consists of one-way analysis of variance (ANOVA). *p* < 0.05 was regarded as a significant difference via Duncan’s test. Pearson’s correlation analysis was applied to explore the correlation among different physicochemical indicators. The results were plotted using Origin (version 22.0, OriginLab Corp., Northampton, MA, USA).

## 3. Results and Discussion

### 3.1. Moisture Content, WAI, and WSI

**Figure 1:**
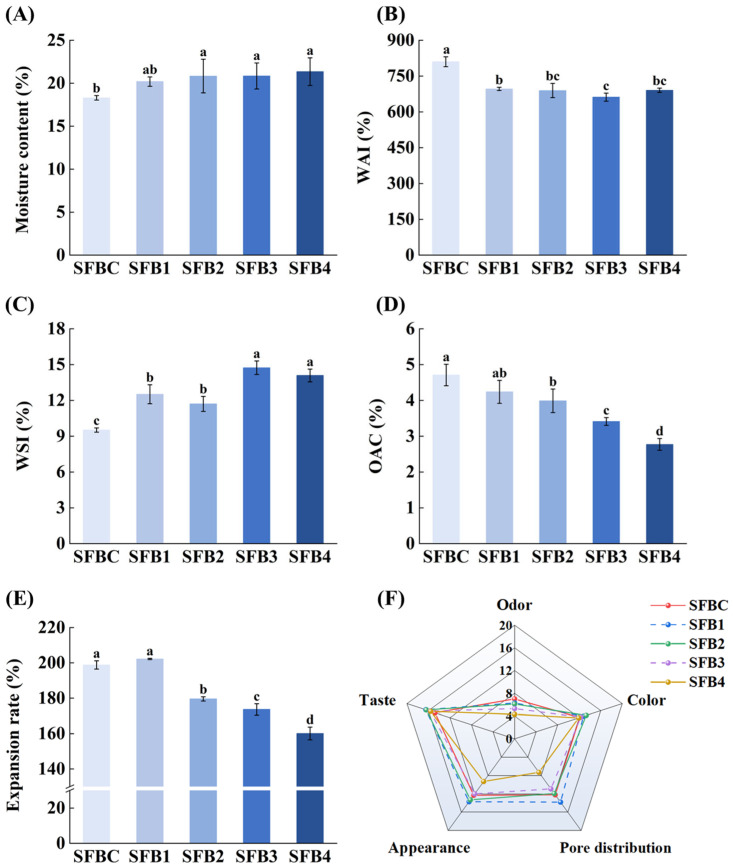
Effect of various proportions of surimi addition on the moisture content (**A**), WAI (**B**), WSI (**C**), OAC (**D**), expansion rate (**E**), and sensory evaluation (**F**) of extruded SFB. Values are presented as mean ± SD (*n* = 3). Different lowercase letters in the same column indicate significant differences (*p* < 0.05).

**Figure 2:**
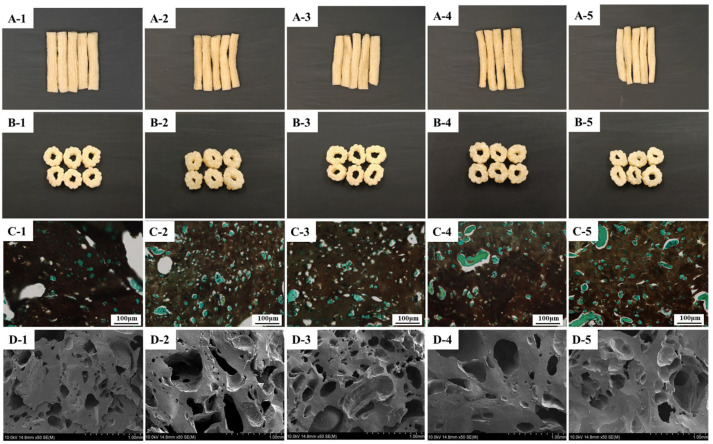
The appearance of surface (**A**) and cross-section (**B**), and the microstructure of optical microscopy (**C**) and SEM (**D**) (with 50× magnification) of the extruded SFB with various proportions of surimi addition. Note: Different numbers represent different samples (1: SFBC, 2: SFB1, 3: SFB2, 4: SFB3, 5: SFB4).

The moisture content is a critical indicator assessing the quality of puffed flour products, as illustrated in Figure 1A. The initial moisture content of SFBC was 18.31 ± 0.27%. With the addition of surimi, the moisture content of the blended system significantly increased (*p* < 0.05). In particular, the SFB4 samples had a 16.6% higher content than the control group. On the one hand, this phenomenon might be attributed to the substantial temperature difference between the interior and exterior of the barrel in the single-screw extruder during processing. With the incremental addition of surimi, the proportion of starch in the blended system decreased gradually, contributing to an increase in encapsulation between flour powder and fish protein, which reduced the degree of water vaporization from the blended system [22]. On the other hand, surimi itself has a highly hydrated gel network and excellent water-binding ability. With the amount of surimi added increasing, the water retention in the extrudate can be enhanced, which might be due to the hydrophilicity of fish protein [23]. Moreover, the expansion pores were significantly enlarged (see Figure 2D) after more surimi was added, indicating that the cavities in the pore structure could retain more water and increase the moisture content.

The WAI reflects the binding capacity between dough and water, which plays a crucial role in food formulations, the expansion rate, and the consistency of the final products [15]. As shown in Figure 1B, the SFBC samples presented the highest WAI of 810%. When 10% surimi was added (SFB1 samples), the WAI results were reduced by 14.04% compared with those of SFBC. Nevertheless, there was no significant decrease in the WAI with the addition of different amounts of surimi (*p* > 0.05), indicating that the inclusion of surimi had a positive effect on lowering the gelatinization potential. The WAI is closely associated with the concentrations of carbohydrates, proteins, and other hydrophilic constituents in flour. As the proportion of surimi increased, protein interactions became more compact, leading to a reduction in exposed hydrophilic groups during the extrusion process, thus diminishing the water-binding capacity of the dough. Moreover, WAI is influenced by alterations in protein conformation (e.g., secondary structure) and the interaction intensity between polar amino acids and water molecules [14].

As illustrated in Figure 1C, with the accumulation of surimi, the WSI gradually increased, from the initial value of 9.52 ± 0.18% (SFBC) to the maximum amount of 14.74 ± 0.57% (SFB3). This could be attributed to the extrusion puffing process, where an increased ratio of surimi addition facilitates the formation of hydrogen bonds and other intermolecular connections. Hence, more soluble polymers and small-molecule substances were generated. Moreover, both the internal and external molecular structures of cooked blended SFB were disrupted with increasing surface area, which contributed to a gradual increase in the water solubility of the samples [24]. Nevertheless, when the addition ratio was 4:6 (sample SFB4), the friction force of the SFB in the barrel decreased with less shearing effect, resulting in the incomplete release of the soluble fractions and a slight reduction in the water solubility of the extruded SFB.

### 3.2. OAC

The OAC is a crucial parameter for evaluating the ability of extruded SFB to retain oil. It is generally believed that the higher the OAC of extruded SFB is, the slower the oil loss rate within the internal pores of the SFB, reflecting better mouthfeel and juiciness [25]. The OAC of extruded SFB clearly decreased gradually with the addition of surimi, as shown in Figure 1D. The SFBC group presented the highest OAC (4.71 ± 0.30%), followed by the SFB1 group (4.24 ± 0.32%). However, no significant difference was observed between these two groups (*p* > 0.05). When the surimi content exceeded 20%, the OAC was significantly lower than that of the control group (*p* < 0.05). The OAC of extruded SFB was closely related to its expansion ratio and internal pore structure, suggesting that adding an appropriate amount of surimi increased the internal pore distribution, formed a dense and ordered starch–protein network structure, enhanced the contact surface area, and increased the expansion ratio (Figure 1E). The presence of this highly crosslinked starch–protein network can provide a physical barrier that inhibits oil absorption [26]. On the one hand, with the increasing addition of surimi, high-temperature puffing induced cross-linking and aggregation of fish protein and glutenin and produced a protective barrier coating of starch particles, inhibiting oil absorption and reducing the OAC of SFB [27]. On the other hand, excessive surimi addition contributed to a more compact gluten network structure, which increased the density of the extruded SFB. During extrusion, the cell walls might rupture, and bubbles fail to expand adequately, leading to smaller pores. Consequently, oil molecules were less able to penetrate the product interior, which reduced the OAC [28].

### 3.3. RER

The expansion rate of the porous structure in the starch-based products primarily affected the starch structure, as shown in Figure 1E. With an increasing proportion of surimi, the RER initially increased, where the maximum RER was observed in the SFB1 samples (202.20 ± 0.36%). However, the addition of excess surimi led to a notable reduction of 20.81% in the RER of the SFB4 samples compared with that of the SFB1 samples (*p* < 0.05). During high-temperature extrusion, a stable protein network structure formed owing to thermal denaturation. Simultaneously, starch underwent a phase transition followed by gelatinization and coagulation. Under increased pressure, the starch granules gradually absorbed moisture and swelled. When subjected to rapid decompression, the starch granules ruptured, and the interior moisture evaporated, ultimately forming a reticular porous structure [1]. Owing to the addition of surimi, a denser gluten network formed within the extruded SFB, which hindered internal pore formation. Moreover, surimi elevated the starch gelatinization temperature and interfered with its integration into the gluten matrix, reducing the expansion capacity of the SFB samples [29].

### 3.4. Sensory Evaluation

The impact of the addition of various amounts of surimi on the sensory evaluation of the odor, color, pore distribution, appearance, and taste of extruded SFB is illustrated in Figure 1F. Compared with SFBC, the incorporation of surimi had a negative effect on the odor of extruded SFB, which gradually decreased as the surimi content increased. Nevertheless, the undesirable impact was not statistically significant between the SFB1 and SFB2 groups. The color, pore distribution, appearance, and taste scores initially increased but then decreased with increasing proportion of surimi accretion, which corresponded to the trend observed for the expansion rate (Figure 1E) and microstructure (Figure 2C,D). The highest results for pore distribution, appearance, and taste were achieved in the SFB1 samples, representing increases of 13.11, 11.38, and 11.49%, respectively, compared with those of the control group. The differences between the SFB1 and SFB2 groups were minimal. Notably, the excessive addition of surimi (SFB4) resulted in the worst results among these factors, and the scores for odor, color, pore distribution, and appearance reached their minimum values, decreasing by 38.57, 1.65, 40.16, and 24.39%, respectively, compared with those of the SFBC. These findings verified that the mouthfeel and non-stickiness of extruded SFB could be improved by adding surimi, which confirmed the results of WAI and textural properties. However, excessive surimi intensified the fishy odor and reduced the expansion rate, consequently lowering the sensory score. In conclusion, the sensory attributes were most favorable in the SFB1 and SFB2 groups, indicating that moderate surimi addition positively enhanced the sensory characteristics of extruded SFB.

### 3.5. Color

The color changes in extruded SFB caused by different surimi addition ratios are presented in Table 1. As the amount of surimi increased, the L*, b*, and h_ab_* values of SFB gradually decreased, whereas the a*, Cab*, and ΔE values increased progressively. The L* value for the SFB4 samples reached its lowest point (89.24), significantly lower than that of the control group (92.15) (*p* < 0.05); the b* value similarly decreased from −0.44 to −4.65, representing a 90.53% reduction compared with that of the control group. This change was subject to the synergistic effect of Maillard browning and starch caramelization reactions [1]. A higher L* value was achieved when the starch particles remained in their original white granular state and were uniformly dispersed throughout the matrix [30]. Additionally, the decrease in L* could be attributed to the darker inherent color of the surimi paste than that of the flour powder. Alterations in raw material proportions and conformational changes in the mixed network could impact the overall color of extruded SFB [31]. However, with the addition of surimi having a minor negative effect on the final color peculiarities of extruded SFB products, the L* value of the SFB1 samples merely decreased by 2.22% compared with that of the SFBC samples, along with a 6.5% increase in the ΔE results, which remained within the range of consumer sensory acceptance. The absorption of visible light by the network structure formed between surimi and flour components might contribute to these results [8].

### 3.6. TPA

The textural properties of the different extruded SFB groups are shown in Table 2. The increase in the proportion of surimi contributed to a gradual decrease in hardness, springiness, and chewiness indicators compared with those of the control group. In particular, the SFB4 samples presented the lowest values of 29.50 ± 2.66 N, 0.86 ± 0.06, and 11.55 ± 0.83 mJ for the abovementioned indices. This variation tends to align with previous findings, which reported that the hardness of starch–protein systems decreased with increasing fresh water fish muscle content in fish noodles and starch-surimi gels [11,30]. As the proportion of surimi increased, the overall protein content within the mixed system accumulated. The enhanced water retention capacity of fish protein led to greater water binding within the protein network. This high-moisture environment reduced intermolecular interactions, resulting in a decline in both hardness and chewiness indicators [32]. On the one hand, flour powder was utilized to facilitate protein expansion, leading to a looser internal pore structure in the extrudate, thus reducing its textural properties [33]. On the other hand, the hydrogen bonding between proteins and starch might inhibit starch recrystallization and moisture redistribution, further decreasing the hardness of the extruded SFB [34]. Furthermore, the observed reduction in hardness and chewiness could be related to the increasing moisture content as surimi was added (Figure 1A), which was assumed to be negatively correlated with moisture content and textural properties.

### 3.7. Appearance and Microstructure

The apparent shape and cross-section of the different extruded SFB samples are shown in Figure 2A,B. The SFBC and SFB1 groups were in an objective puffed state with thick and elastic body walls, which was consistent with the expansion rate results (Figure 1E). When the amount of surimi added exceeded 20% in extruded SFB samples, the surface was not plump, and the body wall became thin; therefore, the status of the products gradually declined.

The microstructure of extruded SFB samples was observed via optical microscopy with iodine–potassium iodide staining (Figure 2C), where starch and protein substances were stained black and green, respectively. The control group (SFBC) exhibited an expanded microstructure characterized by larger pores with an uneven distribution, along with an irregular dispersion of proteins in the extruded SFB body wall. In contrast, the SFB1 and SFB2 samples presented the most uniform protein distributions without significant aggregation, and their pore structures were relatively homogeneous. However, when the surimi content exceeded 30%, protein aggregation became evident, resulting in an uneven protein distribution and a reduced puffing rate.

SEM images revealed a porous architecture in the extruded SFB (Figure 2D). Compared with the control and SFB2 samples, the SFB1 samples formed the largest holes in the internal structure of the SFB, with more small pores in the body wall. When the proportion of surimi surpassed 30%, there were still a few large pores within the surimi–flour mixed system. The thickness of the SFB body wall increased, accompanied by fewer small pores. Typically, flour-based products with the desired textural properties have larger pores and thinner body walls, which makes the extruded substances conducive to chewing [35]. With an appropriate proportion of surimi, the moisture in the samples evaporated completely during the puffing process, which formed more gas chambers, thus resulting in an excellent internal structure [36]. As the addition of surimi increased, the percentage of starch content decreased gradually, resulting in a reduced expansion rate and a thicker inner body wall. Furthermore, during continuous high-temperature extrusion, moisture evaporated prematurely, which contributed to insufficient expansion momentum and uneven gas chamber formation [37]. These changes eventually led to changes in the microstructure of the extruded SFB samples.

### 3.8. DSC

**Figure 3:**
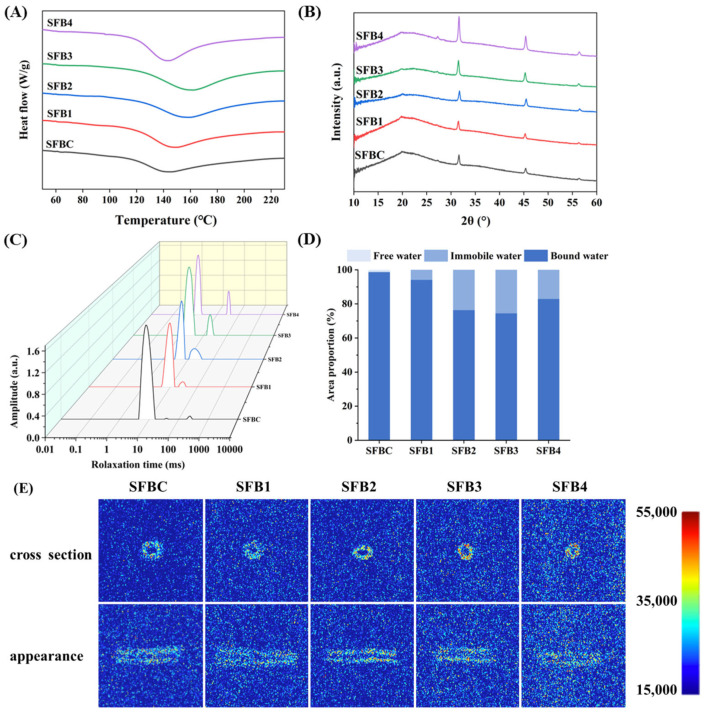
The DSC (**A**), XRD (**B**), LF-NMR relaxation time curves and peak areas proportion (**C**), relative content of different water states (**D**), and pseudo-color images (**E**) of extruded SFB with various proportions of surimi addition.

The DSC curves of all the extruded SFB samples showed a single endothermic peak, suggesting that diverse proportions of surimi do not alter the crystal structure of the mixed system (Figure 3A). The thermodynamic parameters of the extruded SFB samples were further analyzed and are presented in Table 2. With the increasing surimi ratio in the mixed system, both the T_0_ and T_p_ indicators initially increased but subsequently decreased, reaching maximum values of 126.05 ± 1.99 °C and 164.74 ± 3.68 °C, respectively. The △H index showed a continuous upward trend. In particular, the ∆H of the SFB3 samples was significantly greater than that of the other samples, with a value of 124.83 ± 5.26 J/g. These changes in thermodynamic properties are associated with the starch content, moisture content, and presence of exogenous additives, such as dietary fiber and protein [30,38]. As the amount of added fish protein increased, the thermal stability of the system improved, requiring greater energy and higher temperatures to break hydrogen bonds [39,40]. A larger ∆H indicates more extensive recrystallization, which implies stronger interactions between starch chains and a greater degree of starch aging [41]. Therefore, adding surimi at ratios exceeding 20% might significantly increase starch aging [30]. The decrease in Tp might be attributed to excessive surimi addition to the SFB4 samples, leading to protein aggregation and consequently weakening the binding capacity between protein and starch molecules [12].

### 3.9. XRD

XRD is an effective method for evaluating and quantifying the long-range crystalline structure of starch. The crystalline forms associated with starch are primarily categorized into A-, B-, C-, and V-type structures, where the C-type represents a hybrid of A- and B-form crystals [42]. The XRD patterns of the extruded SFB samples are presented in Figure 3B. A prominent diffraction peak appeared at 2θ = 20°, which corresponded to the characteristic V-type crystalline structure of starch [43]. This structural feature arose from the formation of single-helix complexes between amylose and small molecules, which are commonly referred to as V-type amylose complexes. The appearance of this peak can be attributed primarily to the expansion of the amylose–surimi complexes [1,44]. Moreover, the addition of surimi at different scales did not alter the peak width, suggesting that surimi does not influence the overall crystal type of the extruded SFB. However, when the amount of surimi added was increased to 20 and 30%, the intensity of the diffraction peak at 2θ = 20° was significantly reduced. This reduction might lead to an accumulation of hydrophilic binding sites within surimi, which enhances hydrogen-bonding interactions with water and hydroxyl groups in the flour matrix. These interactions can promote the formation of more methodical structures, thus decreasing the amorphous content [45]. The relative intensity of the diffraction peak near 2θ = 31° increased. This phenomenon might be attributed to the reduced aggregation of surimi proteins at higher concentrations under high-temperature extrusion conditions, which ultimately facilitates the formation of a more organized helical structure within the mixed system [46]. Furthermore, increasing the amount of added surimi enhanced the recrystallization degree of the system. This occurred because the elevated protein content led to increased encapsulation of starch particles, thereby restricting water absorption and reducing the likelihood of disruption to the crystalline structure [1].

### 3.10. LF-NMR Relaxation and MRI Analyses

Moisture migration is closely related to the quality of flour-based products. Both the physical state and spatial distribution of water can influence its physicochemical properties during processing [47]. The T_2_ relaxation curves of the extruded SFB with varying proportions of added surimi are presented in Figure 3C. The control group presented three distinct peaks in the T_2_ curve. However, upon the addition of surimi, the T_21_ relaxation time gradually decreased, whereas the T_23_ peak (>100 ms) disappeared, indicating that tightly bound water and immobilized water became more prominent in the surimi-containing samples. These results suggested that the synergistic interaction between surimi proteins and starch promoted the formation of a more ordered gel network, thus enhancing the binding of water molecules within the matrix. Additionally, surimi proteins might form hydrogen bonds with water molecules, reducing their mobility [44]. To further quantify the moisture distribution in each sample, the relative proportions of water in different states are shown in Figure 3D. The proportion of bound water (P_21_), corresponding to the T_21_ component, accounted for more than 60% of the extruded SFB, indicating that P_21_ constituted the dominant water fraction. This water is involved primarily in hydration with protein molecules and resides within the microstructure of the system [43]. However, as the surimi content increased, P_21_ first decreased but then increased, whereas the immobilized water (P_22_) showed the opposite trend. These findings indicated that surimi facilitated the transformation of bound water into immobilized water during the extrusion puffing process.

MRI is a powerful tool for visualizing the internal structure of food materials. Proton density-weighted MRI is commonly used to assess the spatial distribution of water and lipids, where image brightness correlates positively with the total content of these components [48]. The dark blue regions represent areas with low water and lipid contents, whereas the red regions indicate high levels of water and lipids in the gel matrix [49]. Generally, high signal intensity and bright regions correspond to bound or immobilized water, whereas low signal intensity indicates free water. As shown in Figure 3E, with the increasing surimi content, the red-colored area expanded, and the overall signal intensity of the extruded SFB increased, suggesting a greater proportion of bound and immobilized water. When surimi was added, proton binding was enhanced, leading to interactions between water molecules and myofibrillar proteins, thus converting free water into bound or immobilized forms [50]. This improvement had a positive impact on the water-holding capacity, which aligned with the changes in moisture content and LF-NMR results.

### 3.11. FTIR

**Figure 4:**
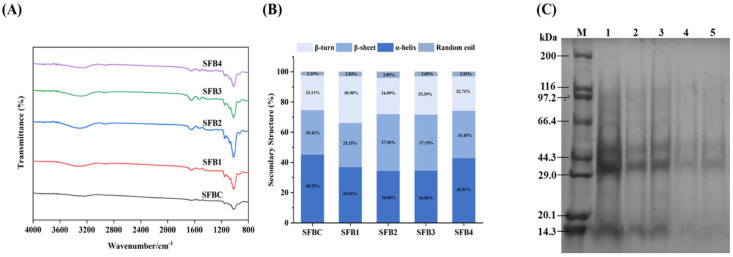
The FTIR (**A**), secondary structure (**B**), and SDS–PAGE (**C**) of extruded SFB with various proportions of surimi addition. Note: Different lowercase letters in the figure indicate significant differences (*p* < 0.05). The vertical line represents the standard deviation (*n* = 3); M represents the protein marker, 1 for SFBC, 2 for SFB1, 3 for SFB2, 4 for SFB3, and 5 for SFB4.

FTIR is an analytical technique that identifies functional groups in substances by measuring the absorption of infrared radiation in a specific wavelength range. It is widely used for structural analysis of diffractive materials such as starch and protein [51]. Figure 4A shows the full-band infrared spectrum of extruded SFB samples in the range of 800–4000 cm^−1^. The positions and shapes of the absorption peaks in the FTIR spectra of the five groups with varying proportions were largely consistent, without new absorption peaks appearing or existing characteristic peaks disappearing. A broad O–H stretching vibration peak was observed between 3500 and 3200 cm^−1^, indicating that increasing the surimi content led to greater hydrogen bond formation due to the cleavage of glycosidic bonds in the mixed system. The peak at 2930 cm^−1^ corresponded to the symmetric stretching vibration of the C–H group [52]. The absorption bands between 1150 and 1000 cm^−1^ reflected strong C–O and C–C stretching vibrations across all five sample groups, suggesting a high degree of hydrogen bonding in the system.

To investigate the effects of the addition of surimi on the secondary structure of extruded flour-based products, we created Figure 4B, illustrating the relative contents of α-helix, β-sheet, β-turn, and random coil structures. As the surimi content increased, the β-sheet, β-turn, and random coil contents initially increased but then decreased; nevertheless, the α-helix content exhibited the opposite trend. Among these samples, SFB1 presented the highest β-turn content of 30.90 ± 1.50%. Compared with that of the control sample, the α-helix content of the SFB2 sample reached its lowest value, showing a 23.79% decrease, whereas the β-sheet content increased to 37.56 ± 0.48%. Compared with that in the control group, the α-helical structure in the extruded SFB was partially converted into a β-sheet structure after surimi addition. This suggested that surimi induced protein unfolding, exposing more hydrophobic groups and enhancing the interaction between proteins and starch [53]. The reduction in the α-helical conformation increased the number of active protein sites, which facilitated cross-linking between proteins and flour starch to form a composite gel network with larger pore diameters and thicker pore walls (Figure 2). The secondary structure and spatial distribution of proteins are closely associated with protein polymerization and the development of the gluten matrix (gliadin and glutenin), where higher contents of α-helix or β-sheet conformations generally indicate greater structural stability in flour-based products [54,55]. As reported in a previous study, the addition of soy protein and wheat gluten to the Alaskan pollock surimi-based meat analog system enhanced the degree of protein cross-linking. This effect was particularly pronounced after protein denaturation induced by high temperature, high pressure, and high shear conditions, which exposed hydrophobic regions and hydrogen bonds, thereby promoting protein–protein interactions and facilitating the formation of a more stable protein network structure [12]. In the SFB1 samples, the total content of α-helix and β-sheet structures was lower than that in the other groups, demonstrating a relatively unstable structure, which was more conducive to puffing during processing. This observation aligned with the expansion rate results shown in Figure 1E, indicating that the appropriate addition of fish protein enhanced the puffing performance. However, excessive protein addition stabilized the protein structure of the extruded product and had a negative effect.

### 3.12. SDS–PAGE

As shown in Figure 4C, the SDS–PAGE pattern was used to illustrate the molecular weight distribution of the extruded SFB with different additions of surimi, which served as an indicator for assessing the thermal polymerization characteristics of proteins [26]. The protein molecular weights of the SFBC samples were approximately 44.3, 29, and 14.3 kDa, consistent with the previously reported wheat flour SDS–PAGE pattern [27]. When surimi was added, no distinct myosin heavy chain (MHC) bands were detected. This absence suggested that molecular interactions between MHC molecules or between MHC and gluten led to the formation of complex aggregates, thus altering the protein structure in the composite system and ultimately forming a cross-linked network structure [45]. Nevertheless, with surimi accumulation, the intensity of these protein bands gradually decreased. This trend indicated that the protein interaction network (involving myofibrillar proteins, gliadin, and glutenin) became progressively more compact, which aligns with the results of the secondary structure analysis (Figure 4B). During extrusion processing, proteins are subjected to combined thermal and mechanical stresses from the heating barrel and shearing screw, leading to structural unfolding and subsequent cross-linking with other flour components to form starch–protein complexes [13]. Therefore, incorporating surimi into wheat flour facilitated the formation of a dense protein network structure; as the surimi content rose, this network became increasingly stable with increased cross-linking, which was not conducive to puffing.

### 3.13. Correlation Analysis

**Figure 5:**
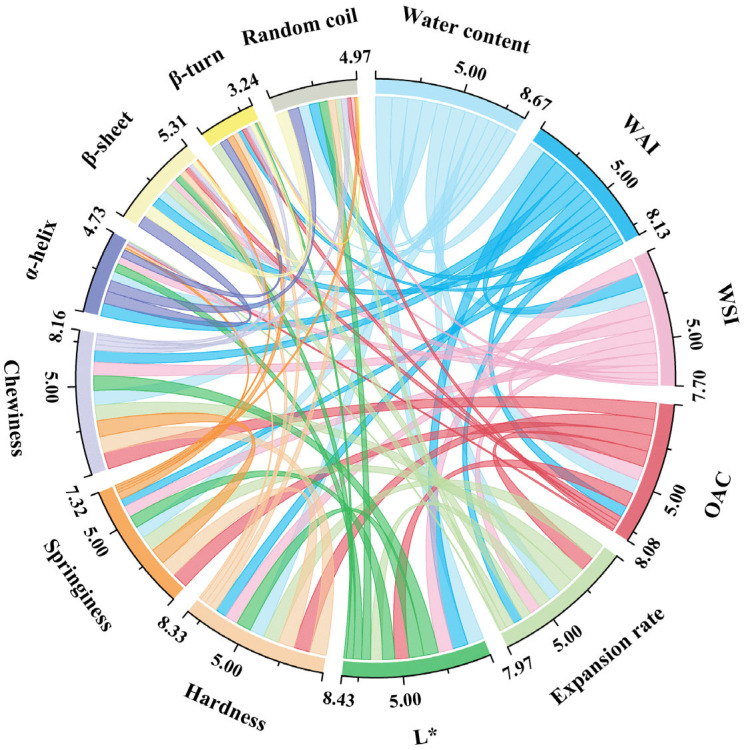
The correlation analysis of different indicators of extruded SFB with various proportions of surimi addition. L*: brightness, WAI: water absorption Index, WSI: water solubility index, and OAC: oil-holding capacity.

To further reveal the influence of each indicator on the quality characteristics of extruded SFB samples, we prepared a correlation chord plot, as presented in Figure 5. Each node in the chart represents an indicator area, where the scale on each node indicates the magnitude of the relationship strength between that indicator and others. Additionally, the width of the connecting chords reflects the strength of the correlation between two corresponding indicators. In descending order of relationship strength values, the indicators that had the greatest influence on the extruded SFB were moisture content (8.67), L* (8.43), hardness (8.33), chewiness (8.16), WAI (8.13), and OHC (8.08). As shown in Figure 5, the moisture content exhibited a significant negative correlation with the WAI (r = −0.93, *p* < 0.05). On the one hand, when the moisture content increased beyond a certain threshold, excess water occupied voids or active sites that could adsorb additional water molecules, thus reducing the water-absorbing capacity of the surimi–flour mixed system and lowering the WAI results. On the other hand, high moisture might transform the physical structure of the SFB, diminishing its ability to bind water and consequently lowering the WAI. The moisture content synchronously positively correlated with the WSI (r = 0.86), indicating that the dissolution of substances increased with increasing diffusion and solvation of solute molecules. Furthermore, the expansion rate significantly positively correlated with the OHC (r = 0.93, *p* < 0.05), indicating that the expansion process might induce conformational changes in macromolecules, such as proteins and polysaccharides. These groups exhibited higher affinity for lipid molecules, enabling stronger oil binding and thus improving the oil-holding capacity. L* was strongly negatively correlated with moisture content (r = −0.98, *p* < 0.01), implying that as the moisture content increased, the brightness decreased. This phenomenon might result from moisture altering the light scattering and absorption properties within the system. Excessive moisture can increase diffuse reflection inside the food matrix, reducing surface reflectance intensity and hence lowering brightness [12]. Hardness, elasticity, and chewiness are strongly interrelated, which can be explained by their shared dependence on the internal structure and intermolecular interactions within the extruded SFB. A tight network structure typically leads to corresponding changes in these textural properties. There is a complex interplay among α-helix, β-sheet, β-turn, and random coil structures. β-sheets were positively correlated with random coils (r = 0.71) but negatively correlated with α-helices (r = −0.61). This occurred because the formation of protein secondary structures is governed by amino acid sequences and both intra- and intermolecular interactions. Different secondary structures compete during formation; an increase in one structure often restricts the development of another, resulting in specific correlations between them.

## 4. Conclusions

The different amounts of surimi added to wheat flour can effectively improve the quality characteristics of extruded flour-based products. Compared with the control group, the extruded SFB samples had a higher moisture content and WSI, lower OAC, and denser network structure. This indicated that the addition of surimi increased protein cross-linking and the water retention capacity. As the surimi proportion increased, the secondary structure of the protein in the extruded SFB became more stable. Nevertheless, the addition of a low proportion of surimi improved the textural characteristics of extruded flour-based products, especially the textural characteristics. Notably, the texture properties of the SFB1 sample were similar to those of the control group, with a higher expansion rate and β-turn content. These results provide useful information for the multivalue utilization of surimi and offer a theoretical basis for research on surimi–flour mixed systems. On the basis of the interactions among gluten protein, starch, and other substances in flour-based products and myofibrillar proteins, this study presents a novel approach for developing new types of puffed flour foods enriched with animal protein. In future studies, the optimal surimi–flour ratio under varying extrusion conditions, including temperature and screw speed, could be further investigated, along with the long-term storage stability. The objective is to broaden the application of enriched surimi in industrial production and to address consumer demand for nutritious, high-quality, and diverse cereal-based products.

## Figures and Tables

**Table 1:** Effect of various proportions of surimi addition on the color attributes of extruded SFB.

Sample	L*	a*	b*	h_ab_*	C_ab_*	ΔE
SFBC	92.15 ± 0.54 ^a^	5.96 ± 0.05 ^c^	−0.43 ± 0.03 ^a^	−0.07 ± 0.00 ^a^	5.98 ± 0.04 ^d^	7.31 ± 0.01 ^e^
SFB1	90.10 ± 0.43 ^b^	6.00 ± 0.08 ^c^	−0.60 ± 0.06 ^a^	−0.10 ± 0.01 ^a^	6.03 ± 0.08 ^d^	7.82 ± 0.22 ^d^
SFB2	90.07 ± 0.14 ^b^	6.06 ± 0.09 ^bc^	−1.78 ± 0.11 ^b^	−0.29 ± 0.02 ^b^	6.32 ± 0.08 ^c^	8.13 ± 0.04 ^c^
SFB3	89.83 ± 0.19 ^bc^	6.14 ± 0.04 ^ab^	−2.49 ± 0.15 ^c^	−0.39 ± 0.02 ^c^	6.63 ± 0.04 ^b^	8.51 ± 0.09 ^b^
SFB4	89.24 ± 0.13 ^c^	6.21 ± 0.06 ^a^	−4.65 ± 0.26 ^d^	−0.64 ± 0.03 ^d^	7.77 ± 0.11 ^a^	9.75 ± 0.18 ^a^

Note: Values are presented as mean ± SD (*n* = 3). Different lowercase letters in the same column indicate significant differences (*p* < 0.05). h_ab_*: hue, C_ab_*: chroma, and ΔE: color difference.

**Table 2:** Effect of various proportions of surimi addition on the textural properties and thermostability of extruded SFB.

Sample	Texture			DSC		
	Hardness (N)	Springiness	Chewiness (mJ)	T_0_ (°C)	T_p_ (°C)	∆H (J/g)
SFBC	38.34 ± 2.49 ^a^	1.36 ± 0.05 ^a^	32.68 ± 2.40 ^a^	123.68 ± 5.20 ^a^	148.74 ± 11.09 ^ab^	59.87 ± 8.60 ^b^
SFB1	36.14 ± 1.80 ^ab^	1.32 ± 0.09 ^ab^	26.63 ± 2.57 ^b^	122.6 ± 3.27 ^a^	141.74 ± 4.63 ^b^	61.02 ± 4.04 ^b^
SFB2	33.82 ± 3.02 ^b^	1.24 ± 0.10 ^bc^	23.44 ± 1.17 ^c^	126.05 ± 1.99 ^a^	159.20 ± 5.45 ^a^	116.83 ± 4.56 ^a^
SFB3	33.46 ± 1.78 ^b^	1.16 ± 0.04 ^c^	20.46 ± 1.15 ^d^	125.83 ± 2.85 ^a^	157.02 ± 6.90 ^ab^	124.83 ± 5.26 ^a^
SFB4	29.50 ± 2.66 ^c^	0.86 ± 0.06 ^d^	11.55 ± 0.83 ^e^	120.74 ± 3.72 ^a^	142.22 ± 9.51 ^b^	117.83 ± 11.93 ^a^

Note: Values are presented as mean ± SD (*n* = 3). Different lowercase letters in the same column indicate significant differences (*p* < 0.05). T_0_ and T_p_ indicate the onset and peak temperature, respectively. ∆H represents the crystal melting enthalpy.

## Data Availability

The original contributions presented in this study are included in the article/Supplementary Materials. Further inquiries can be directed to the corresponding authors.

## Supplementary Materials

The following supporting information can be downloaded at: https://www.mdpi.com/article/10.3390/foods14162851/s1, Table S1: The formula of SFB with various proportions of surimi addition and the moisture, protein, and starch content of the dough.
