# Supplementary material for: Effects of the Ratio of Alaskan Pollock Surimi to Wheat Flour on the Quality Characteristics and Protein Interactions of Innovative Extruded Surimi–Flour Blends
Source: Foods. 2025 Aug 17;14(16):2851. doi: 10.3390/foods14162851 (PMC12385230; doi:10.3390/foods14162851)
Supplement: Supplementary file 1 [file foods-14-02851-s001.zip › foods-3793090-supplementary.pdf]

Table S1. The formula of SFB with various proportions of surimi addition and the moisture, protein, and starch content of the dough.

Sample	Surimi (%)	Wheat flour (%)	Water (%)	Salt (%)	Moisture content (%)	Protein content (% dry weight)	Starch content (% dry weight)
SFBC	0	69.0	27.6	3.4	30.38±0.50[a]	9.14±0.14[e]	48.79±0.49[a]
SFB1	7.2	64.8	24.4	3.6	29.24±0.27[a]	10.09±0.05[d]	45.82±0.46[b]
SFB2	15.3	61.1	19.8	3.8	29.62±0.53[a]	11.20±0.16[c]	43.20±0.43[c]
SFB3	24.2	56.5	15.3	4.0	30.31±1.35[a]	12.32±0.21[b]	39.95±0.40[d]
SFB4	34.2	51.3	10.3	4.2	29.57±0.73[a]	13.53±0.28[a]	36.27±0.37[e]

Note: Values are presented as mean ± SD (n = 3). Different lowercase letters in the same column indicate significant differences ($p < 0.05$).
